# Short-term outcome of patients with adult IgA vasculitis: a single-center experience

**DOI:** 10.3389/fmed.2023.1210307

**Published:** 2023-07-17

**Authors:** Alojzija Hočevar, Jaka Ostrovršnik, Vesna Jurčić, Matija Tomšič, Žiga Rotar

**Affiliations:** ^1^Department of Rheumatology, University Medical Centre Ljubljana, Ljubljana, Slovenia; ^2^Faculty of Medicine, University of Ljubljana, Ljubljana, Slovenia; ^3^Institute of Pathology, Faculty of Medicine, University of Ljubljana, Ljubljana, Slovenia

**Keywords:** adult IgA vasculitis, follow-up, relapse, persistent urinary abnormalities, mortality

## Abstract

**Background:**

Follow-up data on IgA vasculitis (IgAV) in adults are scarce. We aimed to investigate the outcome of adult IgAV in a well-defined cohort.

**Methods:**

Data from histologically proven patients diagnosed between January 2010 and July 2022 with at least a 3-month follow-up were analyzed. The frequency and type of relapses and information on kidney function were extracted. Risk factors for IgAV relapse and decline in renal function were studied using the Cox hazards regression analysis. Mortality in IgAV was assessed using the Kaplan–Meier analysis and the standardized mortality ratio (SMR).

**Results:**

In total, 265 patients were followed for a median of 24 months. At baseline, 38.9, 29.8, and 44.5% had articular, gastrointestinal, and renal involvement, respectively. Initially, 189 (71.3%) patients received systemic glucocorticoids, and 32 (12.1%) patients received an additional immunomodulator. During follow-up, 42 (15.8%) patients relapsed. Relapses were more common in younger patients (HR 1.03 [95%CI 1.01–1.05]) and those without baseline glucocorticoid treatment (HR 3.70 [95%CI 2.0–6.67]). Furthermore, 74 (27.9%) patients had persistent abnormal urinalysis and a substantial (≥20%) decline in glomerular filtration rate (eGFR) was recorded in 41 (15.5%) patients. The factors associated with persistent abnormal urinalysis were an absence of IgAV joint involvement and baseline immunomodulatory treatment. Pre-existent chronic kidney disease and heart failure were associated with eGFR decline. The overall SMR was 1.4 (95%CI 1.14–1.71) compared to the Slovenian general population.

**Conclusion:**

IgAV relapses occurred in 15% of patients, with younger patients with symptomatically managed IgAV experiencing it more frequently. Heart failure emerged as a predictor of persistent abnormal urinalysis and a decline in eGFR. Adults with IgAV had increased mortality compared to the general population.

## Highlights

- Relapses affected 15% of adults with IgA vasculitis.
- Younger adults with symptomatically treated IgA vasculitis were more prone to relapse.
- A quarter of adults with IgA vasculitis showed persistent abnormal urinalysis during follow-up.
- Concurrent heart failure had an important impact on the prognosis of adult IgAV.

## Introduction

Immunoglobulin A vasculitis (IgAV) represents a small vessel leukocytoclastic vasculitis characterized histologically by predominant IgA deposits in the inflamed vascular wall and clinically by skin, joint, gastrointestinal, and renal involvement (1).

IgAV is not uncommon in adults (2); nevertheless, its characteristics and management are less well understood compared to the pediatric disease form. Studies have shown that adults have more severe skin and kidney involvement during acute IgAV compared to children (3). Also, adults have a poorer renal prognosis (4–7). In a large French retrospective study, almost 40% of adult patients exhibited moderate to severe renal insufficiency after a median follow-up of nearly 15 years, and only 20% maintained normal renal function (8). In a large Chinese study limited to patients with biopsy-proven IgAV nephritis and a median follow-up of 4.5 years, Huang et al. found a 4.6% progression rate toward end-stage kidney disease (9). The clinical factors associated with poor renal prognosis were similar in both studies: laboratory baseline proteinuria and renal insufficiency, and histologically, the degree of interstitial fibrosis and glomerular sclerosis predicted poor renal outcome (8, 9). Interestingly, Baumrin et al. recently reported that minimal urinary abnormalities at IgAV diagnosis also heralded an increased risk for long-term renal impairment compared to no abnormalities (10). As for IgAV relapses, they are also common in adults (6, 11). Gazel et al., in a retrospective multicentre study, reported a 15% relapse rate during a median follow-up of 15 months (11), and Kang et al. recorded a 20% relapse rate (6). In addition, Batu et al. found persistent haematuria as a factor associated with relapses in adults (7).

An overall unfavorable prognosis of adult IgAV is suggested by IgAV patient survival studies. A study from the United States in patients aged over 50 years found a 7-fold increase in mortality compared to the general population (3). Additionally, a recent Australian study has shown that comorbidities predicted premature death in hospitalized IgAV patients (12).

Existing follow-up studies in adults were generally retrospective, predominately focused on the renal outcome, and rarely included the impact of comorbidities on the outcome. Thus, we aimed to investigate the disease outcome and to determine the potential predictors of relapsing IgAV, renal prognosis, and survival in our well-defined prospectively diagnosed and followed cohort of unselected adult IgAV patients.

## Methods

### Setting and patient selection

This follow-up study was conducted in the Department of Rheumatology, University Medical Centre Ljubljana, Slovenia. In this study, we included adults (i.e., individuals aged ≥18 years) diagnosed for the first time with IgAV between January 2010 and July 2022 and who were followed up for at least 3 months. All included patients had histologically proven (by skin and/or renal biopsy) IgAV and fulfilled the EULAR/PRINTO/PRES classification criteria for IgAV (13).

### Baseline demographics, clinical assessment, and treatment of IgAV

At our unit, we established an outpatient clinic and a protocol for the follow-up of patients with IgA vasculitis. Patients' baseline demographics, comorbidities, IgAV baseline clinical data and therapy, and comorbidities were collected. At diagnosis, all patients underwent a detailed clinical and laboratory work-up as described previously (14). Table 1 provides a summary of definitions of parameters used to clinically characterize IgAV, including the definition of the extent of purpura and renal and gastrointestinal involvement.

**Table 1:** Definitions for the baseline evaluation of IgA vasculitis.

**Purpura**	
Localized	Vasculitic lesions present only below the waistline
Generalized	Vasculitic lesions extend above the waistline
**Renal involvement**	
Haematuria	>5 red blood cells per high power field or red blood cell casts in the urinary sediment or hemoglobinuria ≥2+ on the dipstick
Macrohaematuria	>1,500 red blood cells/mm^3^ of urine
Proteinuria	Protein/creatinine ratio in spot urine sample >30 mg/mmol or urine protein excretion >300 mg/day
Severe	Nephrotic or nephritic syndrome with acute worsening of renal function, defined as either an increase in serum creatinine concentration or a decrease in the glomerular filtration rate estimated by the MDRD-4 >25% from the patient's baseline
GIT involvement	New onset of diffuse abdominal pain or gastrointestinal bleeding
Severe	Bloody diarrhea, ileus, or bowel perforation

GIT, gastrointestinal tract; MDRD-4, Modification of Diet in Renal Disease, 4 variables.

### Follow-up of IgAV

#### Relapses and persistent abnormal urinalysis

Follow-up visits were scheduled 2 to 4 weeks, 3, 6, and 12 months after IgAV diagnosis, and yearly thereafter. If the disease worsened, additional visits were arranged. At each visit, the clinical (cutaneous, gastrointestinal, and articular) and laboratory (renal function and urine analysis, including assessment of daily proteinuria) activities of IgAV were evaluated. Based on our premise that manifestations of the initial IgAV episode would significantly improve or subside in the first 3-month period after diagnosis (and baseline treatment), the following definitions of terms were established: (1) Persistent abnormal urinalysis was defined as persistent haematuria (>10 × 10^6^ red blood cells/L) and/or proteinuria (defined as a protein to creatinine ratio of >30 mg/mmol) on two or more consecutive occasions. (2) A substantial worsening of renal function during the follow-up was defined as an eGFR decline of >20% from baseline. (3) IgAV relapse was defined as a reappearance of clinical or laboratory symptoms and/or signs of IgAV ≥3 months after initial presentation. A relapse included the recurrence of skin purpura with or without concurrent articular, gastrointestinal, and/or renal involvement and the isolated worsening of markers of renal involvement, with increasing haematuria and/or proteinuria with or without the concurrent deterioration of renal function (compared to previous evaluation).

Gastrointestinal involvement at IgAV relapse was defined by abdominal angina with/without gastrointestinal overt or occult bleeding, or diarrhea developing in parallel with skin or renal relapse.

#### Mortality of IgAV patients during follow-up

To evaluate mortality, a censor date, 22 December 2022, was selected. Patients were followed until death or the censor date, whichever came first. Mortality was assessed using the Kaplan–Meier analysis (15). We compared IgAV mortality with the mortality of the age- and sex-matched Slovenian population obtained from the Department of Demographic and Social Statistics at the Statistical Office of the Republic of Slovenia^1^ We analyzed causes of death in individual patients and risk factors of mortality in the IgAV population.

### Statistical analysis

The results were expressed as medians and interquartile ranges (IQRs) for non-normally distributed variables, as average with standard deviation (SD) in normally distributed metric variables, or as absolute numbers and percentages for categorical variables. Two-tailed Fisher's exact test was used to compare categorical variables. We used the Cox regression analysis to evaluate risk factors for the relapse and decline in renal function. Multiple logistic regression was used to investigate predictors of persistently abnormal urinalysis. In the prognostic model of IgAV relapses, 11 variables from demographic factors, disease characteristics, and treatment were included (patient gender, age, constitutional symptoms (weight loss or fever), the extent of skin purpura (above or below the waistline), necrotic skin purpura, articular symptoms (arthritis or arthralgias), bowel involvement, renal involvement, and treatment with glucocorticoids and other immunomodulators). Variables were selected *a priori* based on their perceived clinical relevance. To evaluate predictors of persistent abnormal urinalysis and decline in renal function, six comorbidities, namely, arterial hypertension, hyperlipidemia, heart failure, diabetes mellitus, chronic kidney disease (defined as eGFR < 60 ml/min), and obesity (defined as body mass index ≥30 kg/m^2^), were also included. Among laboratory markers, C-reactive protein and neutrophil to lymphocyte ratio were included in the analyses. The Kaplan–Meier analysis and a standardized mortality ratio (SMR) were used to analyze survival/mortality.

The significance threshold selected in all analyses was set at 0.05.

### Ethics committee approval

The study was approved by the Slovenian National Medical Ethics Committee, Number 159/07/13. At diagnosis, patients provided written consent to the use of their demographic and disease data for the study.

## Results

### Baseline demographics, comorbidity data, IgAV manifestation, and treatment data

During the 127-month observation period, we newly diagnosed IgAV in 362 patients (59.4% men, median [IQR] age 64.2 [45.4; 76.2] years). Of these, 265 patients had a follow-up period of more than 3 months and represented our studied population, and 97 patients were either followed for < 3 months or were lost to the follow-up. These patients were excluded from further analysis. The characteristics of the studied IgAV population including the prevalence of selected comorbidities and presentation features of IgAV are presented in Table 2. Briefly, there were 156 (58.9%) men, the median (IQR) age at diagnosis was 61.6 (44.6; 74.2) years, and all had histologically verified IgAV (skin biopsy positive in 265 patients; in addition, 14 out of 265 patients had a kidney biopsy, with IgA glomerulonephritis proven in all biopsied cases).

**Table 2:** Characteristics of patients at IgAV diagnosis.

**Characteristics**	**No (%)**	**Characteristics**	**No (%)**
**Demographics**		Skin biopsy/positive	265/265 (100%)
Men	156 (58.9%)	Renal biopsy	14 (5.3%)
Age (years)^*^	61.6 (44.6; 74.2)	**Comorbidities**	
Active smokers	58 (21.9%)	Arterial hypertension	117 (44.2%)
**IgAV features**		Diabetes mellitus	46 (17.4%)
Symptom duration time (days)^*^	8 (5; 16)	Pre-existent renal failure^&^	36 (13.6%)
Constitutional symptoms	38 (14.3%)	BMI (kg/m^2^)^*^	28.3 (23.9; 31.8)
Purpura above waistline	135 (50.9%)	Hyperlipidemia	63 (23.8%)
Necrotic purpura	112 (42.3%)	Heart failure	33 (12.5%)
Skin limited IgAV	75 (28.3%)	**Treatment of IgAV**	
Articular involvement	103 (38.9%)	Systemic glucocorticoids	189 (71.3%)
Arthritis	42 (15.8%)	Cyclophosphamide	24 (9.1%)
Gastrointestinal involvement	79 (29.8%)	Mycophenolate mofetil	2 (0.8%)
Severe gastrointestinal involvement^$^	20 (7.5%)	Rituximab	3 (1.1%)
Renal involvement	118 (44.5%)	Hyperimmune gammaglobulins	11 (4.2%)
Severe renal involvement ^#^	30 (11.3%)	Plasma exchange	2 (0.8%)
Elevated serum IgA	102/214 (47.7%)	Colchicine	3 (1.1%)
BVAS-3 ^*^	8 (3, 14)	Dapsone	2 (0.8%)

^*^median (IQR); ^&^eGFR < 60 ml/min/1.73 m^2^; BVAS-3, Birmingham vasculitis severity score version-3; BMI, body mass index; ^$^bloody diarrhea, ileus or bowel perforation; ^#^nephrotic or nephritic syndrome with acute worsening of the renal function.

Patients were managed in line with our common local practice; however, final treatment decisions were left up to the treating physician. IgAV remitted spontaneously in 55 (20.7%) patients, and topical steroids were the only treatment in 21 (7.9%) patients. Indications for systemic immunomodulatory treatment were necrotic skin purpura, bowel involvement of any type, and/or severe renal involvement. We treated 189 (71.3.%) patients with systemic glucocorticoids, while 32 (12.1%) patients needed additional immunomodulatory treatment. One patient required transient hemodialysis.

### Follow-up

#### Relapses and persistent abnormal urinalysis

Patients were followed for a median (IQR) of 24.1 (12.5; 55.2) months (ranging from 3.0 to 146.1 months). Overall, 159 (60.0%) patients remained in complete remission (without relapses or persistently abnormal urinalysis) throughout the follow-up period. During the follow-up, 42 (15.8%) patients relapsed. Table 3 provides the frequency and type of relapses in patients relapsing up to three times. Isolated skin relapses were the most frequent relapse type, representing 52.3% of relapse episodes in patients with up to three relapses. All patients with multiple relapses (≥4 relapses) had skin relapses. The median (IQR) time to first relapse was 9.0 (3.9; 22.4) months.

**Table 3:** Frequency and types of relapses in adult IgAV.

**Number of relapses**	**Number (%) of patients**	**Type of relapse in 33 patients with up to three relapsing episodes** ^*^	
1	24 (57.1%)	Skin	23 (52.3%)
2	7 (16.7%)	Skin + kidney	14 (31.8%)
3	2 (4.8%)	Skin + joints	2 (4.53%)
Multiple (≥4)	9 (21.4%)	Skin + joints + GIT	1 (2.3%)
		Kidney	4 (9.1%)

GIT, gastrointestinal tract; ^*^patients with multiple relapses had skin relapse.

Factors associated with relapses of IgAV using the Cox regression analysis were lower patient age (HR 1.03 [95% CI 1.01; 1.05]) and lack of initial systemic glucocorticoid treatment at IgAV presentation (HR 3.70 [95% CI 2.00; 6.67]).

During the follow-up, 74 (27.9%) patients had persistently abnormal urinalysis. Of the 265 patients, 13 (4.9%) patients developed *de novo* persistent abnormal urinalysis during the follow-up. Persistent proteinuria with a protein/creatinine ratio of 30–100 mg/mmol was recorded in 16 patients (6.0%), and 12 (4.5%) patients had a persistent protein/creatinine ratio of ≥100 mg/mmol.

IgAV-related joint involvement and baseline immunomodulatory treatment emerged in multivariate logistic regression analysis as factors independently associated with the persistence of abnormal urinalysis. The presence of IgAV-related joint involvement was protective against the persistence of abnormal urinalysis (OR 0.42 [95% CI 0.23; 0.79], *p* = 0.007), whereas baseline immunomodulatory treatment was positively associated with the persistence of abnormal urinalysis (OR 2.76 [95%CI 1.26–6.03], *p* = 0.011).

An eGFR decline of >20% from baseline was observed in a total of 41 (15.5%) patients; 18 out of 74 patients with persistently abnormal urinalysis (24.3%) developed an eGFR decline of >20%, and 23 out of 191 patients without clinically apparent IgAV-associated renal pathology (12.0%) had an eGFR decline of >20%—corresponding to 8.6 (95%CI 5.1–13.7) cases per 100 patient-years and 3.8 (95%CI 2.4–5.7) cases per 100 patient-years, respectively, and an incidence rate ratio of 2.27 (95%CI 1.16–4.40); *p* = 0.012). Heart failure (HR 2.50 [95%CI 1.16–5.67]) and pre-existing chronic kidney failure (HR 3.71 [95%CI 1.74–7.92]) were associated with eGFR decline during the follow-up in the entire IgAV cohort. When risk factors of eGFR decline were evaluated separately in the group of patients with IgAV renal involvement at baseline, patient age (HR 1.04 [95% CI 1.00; 1.08]), pre-existing eGFR < 60 ml/min (HR 3.66 [95%CI 1.36–9.85]), and persistent abnormal urinalysis (HR 3.19 [95%CI 1.25–8.15]) emerged as significant. Two of our patients developed end-stage kidney disease in association with IgAV during the follow-up.

#### Mortality of IgAV patients

During the follow-up, 48 (18.1%) patients died. The death of one patient was related to IgAV relapse, while cardiovascular diseases, cancer, and infections represented the most frequent causes of death in-−16 (33.3%), 6 (12.5%), and 6 (12.5%) of patients, respectively. Hepatobiliary disease, respiratory failure, trauma/bleeding, and dementia were recorded as the cause of death in three, two, three, and three patients, respectively. In eight (16.7%) patients, the cause of death was not known.

**Figure 1:**
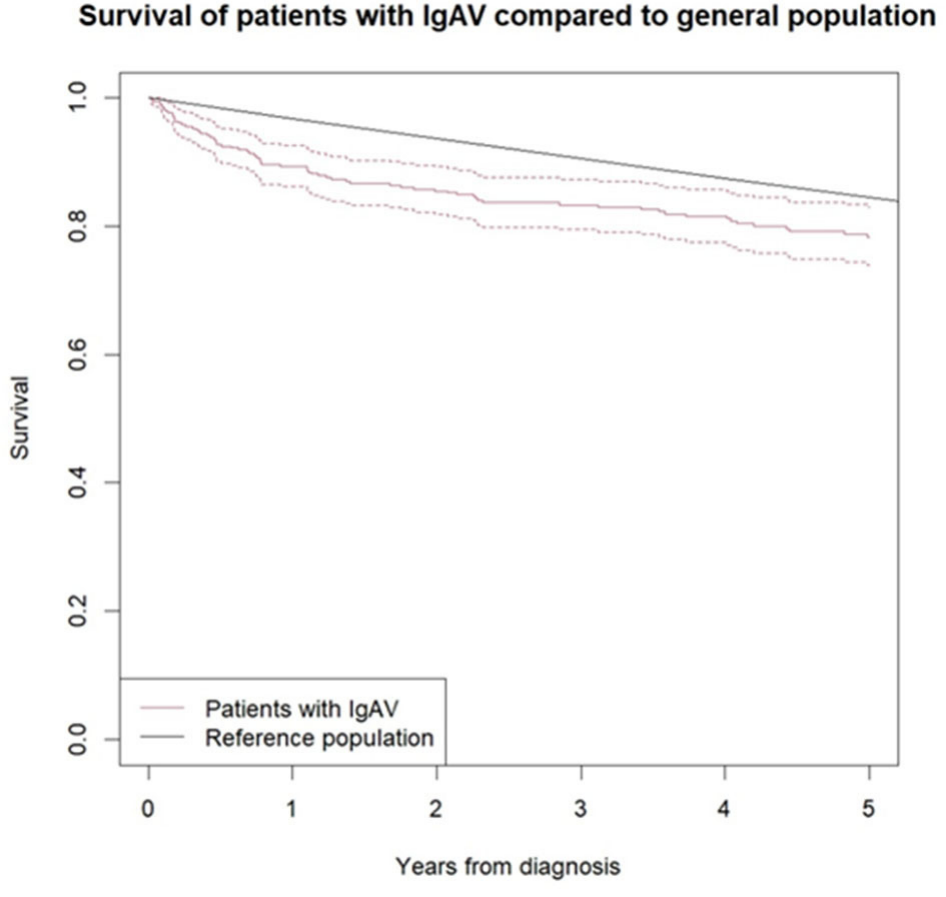
Survival curve of IgAV patients according to Kaplan-Meier analysis compared to sex and age matched general population. Black line represents the survival of general population and red line the survival of IgAV patients, with 95% confidence interval (red dotted line).

The comparison of mortality between the IgAV cohort (during an average observation period of 5.2 years) and the age- and sex-matched general population showed significantly higher overall mortality in the IgAV cohort, with an overall SMR of 1.4 (95%CI 1.14–1.71). Figure 1 shows the Kaplan–Meier survival curves of IgAV patients and the age- and sex-matched general population as a comparator. Excess deaths occurred mainly during the first 2 years of follow-up (Table 4). Five-year survival of our IgAV population was 78% (95% CI 74%; 83%), in adults younger than 51 years, 100% survival; and in adults aged 51 years or more, 67.9% survival. This difference was statistically significant (*p* = 0.01). We found no sex-related differences in survival (*p* = 0.37). Ten-year survival in our patients stood at 65% (95%CI 59–72%). The risk factors associated with increased mortality in IgAV were patient age (HR 1.09 [95%CI 1.06–1.13]), purpura above the waistline (HR 2.14 [95% CI 1.13–4.06]), and pre-existent heart failure (HR 7.37 [95%CI 3.74–14.54]).

**Table 4:** The standardized mortality ratio of adult patients with IgAV.

**Follow-up (years)**	**SMR (95% CI)**	***P*-value**
1	3.79 (2.68–5.20)	< 0.001
2	2.75 (2.05–3.62)	< 0.001
3	2.18 (1.66–2.82)	< 0.001
4	1.84 (1.41–2.35)	< 0.001
5	1.70 (1.33–2.15)	< 0.001
6	1.60 (1.26–1.99)	< 0.001
7	1.53 (1.22–1.90)	< 0.001
8	1.46 (1.17–1.80)	< 0.001
9	1.42 (1.14–1.75)	0.001
10	1.39 (1.12–1.70)	0.002

SMR, standardized mortality ratio.

## Discussion

This study provides a small additional piece toward achieving a better understanding of IgAV in adults. Our data show that 60% of adult IgAV patients reached and remained in remission through the median follow-up of 2 years. In the remaining 40% of patients, relapses or persistence of abnormal urinalysis complicated the disease course. Relapses affected approximately 15% of our cohort. The frequency of recorded relapses was lower compared to the study by Kang et al. (6), who reported a 20% relapse rate during a mean follow-up of 27 months. Although more than half of our patients experienced only one relapse, a substantial number of patients relapsed more frequently. Four or more relapses were recorded in 20% of patients. Only 50% of relapses in adults relapsing < 4 times were limited to the skin. In the remaining cases, various combinations of renal, gastrointestinal, and articular symptoms were recorded, indicating that patients should be thoroughly interviewed and examined also at relapse. When evaluating predictors for a relapsing disease course, we found that older patients had a lower risk of relapse. Our findings are in line with the report from Audemard-Verger et al. (16), who detected the highest frequency of relapses in patients aged < 36 years (27%) and the lowest frequency in patients aged >63 years (11%). Interestingly, also the use of a systemic glucocorticoid at IgAV diagnosis represented an independent protective factor of IgAV relapse in our study. One could assume that younger adults with a baseline less severe disease (not requiring systemic immunomodulatory treatment) are at higher risk of future IgAV relapse. However, unlike Gazel et al. (11), we found no association between acute-phase responses and relapses.

At diagnosis, 45% of our patients had renal involvement. Data from the literature show that renal involvement could manifest for the first time also later, even at IgAV relapse (17). This was the case in nearly 5% of our patients. During the follow-up, we observed a persistent abnormal urinalysis in a quarter of patients. This is far less frequent compared to the study by Kang et al. (6), who observed persistent abnormal urinalysis in almost 60% of adults during the follow-up. However, the Korean population differed from ours already at baseline in the prevalence of renal involvement (79% in the latter). A high prevalence of nephropathy was reported also in a large Spanish IgAV study [58% adults at disease onset, and 84% in fully established disease with nephritic or nephrotic syndrome present in a quarter of patients (25.6%) and renal insufficiency developed in 31% of cases] (18). On the contrary, only 11% of our patients had severe renal involvement at baseline, and by using a different definition, we found a deterioration of renal function in 15% of our patients during the follow-up.

Nevertheless, even mild persisting urinary abnormalities seem to have long-term implications. Baumrin et al. (10) reported that 38% of patients with persisting urinary abnormalities developed long-term renal impairment during a median follow-up of 6 years, compared to only 12% in the group with normal urine analysis. In their study, persisting abnormal urinalysis was more frequently recorded in older patients with known arterial hypertension. In contrast, persistently abnormal urinalysis in our study was associated with the absence of IgAV-related articular involvement and with baseline immunomodulatory treatment. We hypothesize that the latter may “mask” the effect of baseline renal involvement on the persistence of abnormal urinalysis in our cohort, as patients with renal involvement commonly received glucocorticoid and immunomodulatory treatment. An inverse association between joint involvement and the persistence of abnormal urinalysis may point to the existence of various IgAV phenotypes, with the articular phenotype having a more favorable short-term course.

In line with the report by Baumrin et al. (10), we also more frequently detected a significant decline of eGFR in patients with persistent abnormal urinalysis. As additional risk factors of significant eGFR decline in our patients with baseline IgAV renal involvement, increasing patient age, and pre-existing chronic kidney disease emerged. However, when evaluating the entire IgAV cohort, pre-existing heart failure and chronic kidney disease contributed independently to the further decline in renal function. Among investigated comorbidities, concurrent heart failure was also a marker of persistent abnormal urinalysis.

Our results are to some extent comparable to conclusions suggested by García-Porrúa et al., who reported vasculitis relapses, the persistence of haematuria or proteinuria, and the development of renal insufficiency at the last follow-up in 21, 30, and 7% patients, respectively. However, in their study, baseline haematuria, renal manifestations during the disease course, and relapses represented risk factors for renal sequelae (19).

Survival of our IgAV cohort was worse compared to the Slovenian age- and sex-matched general population [an overall SMR of 1.4 (95%CI 1.14–1.71)]. Nevertheless, the 5-year survival rate of our patients aged 51 years or more was better compared to a report from the United States (3) (67 vs. 40%). This difference may be due to a different study period (2010–2022 vs. 1996–2016, respectively), the subsequent overall improvement of general survival over the years, and the inclusion of an unselected IgAV population in our case (with also less severe skin limited disease). Our results regarding survival are comparable to those from Australia, where 72 and 61%, a 5- and 10-year survival rate were observed (12). An analysis of risk factors for increased mortality in our cohort highlighted the extent of skin lesions as an independent risk factor, in addition to patient age and history of heart failure. Patients with skin purpura above the waistline had a decreased survival rate compared to those with skin lesions localized below the waistline. Since we have previously shown that patients with extensive skin lesions more frequently developed IgAV gastrointestinal tract and renal involvement (14, 20), this association may be probable.

Our study has some limitations. One of them is undoubtedly the lack of kidney histopathology data due to the limited number of patients with renal biopsies. At the time of diagnosis, renal biopsy was commonly reserved for patients with rapidly progressive renal failure, nephrotic syndrome, or dubious cases and was not performed routinely in all patients with pathological urine findings, particularly not when the skin biopsy was in accordance with IgAV. Data from a renal biopsy would certainly shed further light on the short- and long-term outcomes of IgAV. Although the study was prospective and included a large number of patients, it was conducted in only one center, and the follow-up time was also limited to a median of 2 years. On the other hand, a monocentric study design could also be an advantage, since in the absence of widely accepted international recommendations for the treatment of adult IgA vasculitis, all our patients received the same therapeutic approach of local practice, and thus, the study outcomes may be easier to interpret. Furthermore, we enrolled an unselected patient population, in view of hospitalization (with both in-patients and out-patients managed), which we believe represents an additional study benefit. As our rheumatology unit is the only referral center for a population of approximately 720,000 adults and serves at the tertiary level a population of approximately three-quarters of the Slovenian population, we believe that we have largely avoided selection bias in the study. In the literature, follow-up analyses were not uncommonly limited to patients with renal involvement or patients who were managed initially in the hospital, potentially predisposing the analysis toward a poorer IgAV outcome.

In summary, our study shows that relapses more frequently affect younger adults with symptomatically managed IgAV. In addition, the data show that, in addition to vasculitis-related factors, comorbidities also have an impact on the prognosis of adult IgA vasculitis.

## Data availability statement

The raw data supporting the conclusions of this article will be made available by the authors, without undue reservation.

## Ethics statement

The studies involving human participants were reviewed and approved by Slovenian National Medical Ethics Committee, No. 159/07/13. The patients/participants provided their written informed consent to participate in this study.

## Author contributions

AH and JO run the IgAV outpatient clinic and contributed to the acquisition of data. VJ as chief pathologist, evaluated the biopsy samples. AH analyzed and interpreted the data and wrote the manuscript. MT and ŽR helped revise the manuscript. All authors meet the authorship requirements and have read and approved the manuscript for submission.

## Footnotes

^1^Available online at: https://pxweb.stat.si/SiStat/sl/Podrocja/Index/100/prebivalstvo.
